# Understanding the Airbnb user continuation intention: The moderating role of perceived risk

**DOI:** 10.3389/fpsyg.2022.929060

**Published:** 2022-08-23

**Authors:** Ahsan Zubair, Rohaizat Baharun, Faiqa Kiran, Muhammad Azeem Abro

**Affiliations:** ^1^Azman Hashim International Business School, University Teknologi Malaysia, Johor Bahru, Malaysia; ^2^Lyallpur Business School, Government College University, Faisalabad, Pakistan; ^3^Department of Business Administration, Sukkar Institute of Business Administration University, Sukkur, Pakistan

**Keywords:** social overload, information overload, perceived risk (PR), continuation intentions, trust in platform

## Abstract

This study evaluates the relationship between diversified relationships established under the umbrella of the Stimuli-Organism-Response (SOR) framework to study the consumer continuation intention of the Airbnb platform from a Malaysian perspective. A web-based survey was conducted among Malaysian Airbnb consumers, and a sample of 303 respondents was obtained. SmartPLS has been used for data analysis. The statistical output of the respondent’s data indicates that social overload and information overload influence consumer continuation intention. Moreover, the satisfaction and trust in the platform partially mediate the relationship between the stimuli and behavioral response. Further, perceived health risk strengthens the negative relationship between continuation and trust in the platform. The theoretical implications include enacting a SOR framework to understand the consumer’s internal state of mind and ability to influence the consumer platform continuation intention. The practical implications suggest that the managers and business owners focus on limiting the social exposure at the host destination and the flow of information from the application.

## Introduction

With the rising popularity of sharing economy in recent years, consumer consumption patterns have changed dramatically. The market has shifted from the conventional business arena to online platforms that have developed novel consumer issues regarding trust in the platform and service satisfaction. Further, the emergence of a pandemic has given rise to risk perception traditionally discussed in terms of monetary loss, safety, and security (Hwang and Choe, 2020; Ventre and Kolbe, 2020). Post-pandemic (COVID-19), the perceived risk is shifting toward the perceived health risk, particularly while encountering a shared platform with other participants, such as Airbnb (Mao et al., 2020). With the introduction of multiple virtual facilities supporting sharing platforms, question marks are raised daily on consumer continuation intention. Hamari et al. (2016) define the sharing economy platform as follows: “The peer-to-peer-based activity of obtaining, giving or sharing the access to goods and services, coordinated through community-based online services.”

Airbnb is recognized as one of the most successful online sharing accommodation platforms (Sthapit et al., 2019). But despite this recognition, Airbnb has been reporting consistent losses since 2017. The platform has reported a $3.9 billion loss in revenue in 2020 alone (Koenig, 2021). Before the pandemic, the company filed a revenue loss of $674 million in 2019 and reported losses in 2018 and 2017 (Koenig, 2021). In Malaysia, the firm faces tough competition from local and international players, such as Agoda, Expedia, Air Asia, OYO Booking.com, and Hyatt. This intense market competition is causing a decline in growth trends. In 2017, the year-on-year (YoY) growth for listings was reported as a 130% increase compared to 2015; the guest listing was 231% better than in 2016. In 2020, eMarketer reported a 60% drop down in Airbnb usage. In 2018, eMarketer issued a report regarding Airbnb’s problematic growth and stated that these trends are not for Malaysia only; the firm is facing similar issues in the United States (eMarketer, 2021). eMarketer reports that Airbnb will add 6.1 million users compared to the estimated 17.6 million (eMarketer, 2021). Moreover, in August 2017, Airbnb’s annual report claimed that each host earned about RM 5,569 per annum (eMarketer, 2021). In 2018, the host suffered a 6% decrease, and their yearly income dropped to RM 5,200 (eMarketer, 2021). Morgan Stanley Alphawise’s survey reports a negative trend and states that the user frequency has fallen to 10% (eMarketer, 2021).

Airbnb business faces unintended financial circumstances when tourists’ continuation intention is compromised. Airbnb provides perishable services with minimum shelf-life. For example, the Airbnb service providers cannot preserve the residential rooms, restrooms, and other assets for another day or sustain them for sale for another time. When Airbnb consumers discontinue or shift to another online service platform, the service provider suffers a decrease in revenue and might face additional costs in terms of maintenance and attracting new consumers (Rama, 2020). Although Airbnb enjoys this cost advantage against classical service providers like hotels and motels, Airbnb suffers a similar repercussion when consumers consider competitors. Besides the hotel, Airbnb enjoys minimum cost to maintain fixed assets. Still, when consumers leave the Airbnb service, the hosts also register to other service platforms to capture more traffic (Dogru et al., 2020).

Losing the active consumer base influences the short- and long-term goals of service providers and stakeholders (Huang et al., 2020). The service providers need to focus on both consumer perspectives of retaining users and investigating the factors leading to continuation intention (Tiamiyu et al., 2020). The literature provides little evidence about exploring Airbnb’s continuation intentions. Liang et al. (2018) examined the continuation of behavioral intention with two forms of satisfaction (transaction-based satisfaction and experience-based satisfaction) and trust (trust in the host and trust in the company) and consequent outcome. Tiamiyu et al. (2020) considered continuation intention as a consumer coping strategy to avoid staying at unfavorable and non-familiar sites causing psychological discomfort influenced by unfair pricing and alternative attractiveness. These Airbnb-based studies have made initial progress in explaining the continuation behavior, focusing on the social, economic, and psychological factors to explain the Airbnb continuation behavior. This situation demands a more holistic approach in the context of Airbnb’s environmental stimulus. These stimuli can influence the consumer’s internal psychological state, adversely impacting the internal mind state necessary for service continuation.

The environmental stimulus is an umbrella statement that explains the motivational factors of Airbnb-based engagement. These motivational characteristics demand further investigation, particularly from a psychological perspective, as they are social and cognitive factors. At the same time, social and information overload are self-explanatory concepts that are considered disadvantages of excessive socialization and unnecessary informational fallow. In online service platforms, such as Airbnb, consumers are highly motivated and free to choose between substitutes if they experience a stressful situation (Huang et al., 2020). If we study the complete cycle of adaptation to continuing IT services, we can predict how consumers nurture such adverse behavioral intentions. Such a reaction considerably impacts consumers and service providers, making it simple to understand the subsequent decisions (Zhai et al., 2020).

The term continuation intention is widely studied in the literature, such as value perception and perceived risk (Liang et al., 2017), perceived risk and brand credibility (Jun, 2020), attitude, subjective norms, perceived behavioral control (Mao and Lyu, 2017), perceived enjoyment (So et al., 2021), experience and traveler personality (Poon and Huang, 2017), affective state and cognitive state (Ahe Ahn and Seo, 2018), utilitarian and hedonic value (Chang I. et al., 2014), and positive and negative emotions (Chen et al., 2015). An et al. (2019) studied the consumer continuation intention in terms of satisfaction influenced by the service quality and value perception. Similarly, the study of Liang et al. (2018) studied the trust in the platform in light of transaction-based satisfaction and experience-based satisfaction. These studies provide evidence of researcher interest in Airbnb’s continuation intention. However, the current body of the literature is unable to constitute the “consumer continuation intention” in relation to service satisfaction and trust in the platform. However, some studies have evaluated the Airbnb characteristics that negatively influence the continuation intention explaining little about the post-consumption stages of the service life cycle. Considering this gap in prior literature about the continuation intention, the current study answers the following questions: “How do the social and information overload influence the consumer trust in platform and service satisfaction, eventually reducing the continuation intention?”

In light of the abovementioned question, the study has certain objectives. First, this study investigates the role of overloads in creating service-related displeasure and their ability to influence the overall continuation intention. Second is the determination of the cognitive and social displeasure at the physical and online services of the platform. Third, the study incorporates the psychological response model Stimuli-Organism-Response (SOR) to measure the consumer continuation intention. Forth, the study will determine the limits to platform crowding and information fallow to the end consumer, as the Airbnb platform is known for its ability to facilitate socialization and information gathering. Lastly, the study presents the findings, implications (managerial and literature), and study limitations.

## Theoretical underpinning and hypotheses development

**FIGURE 1:**
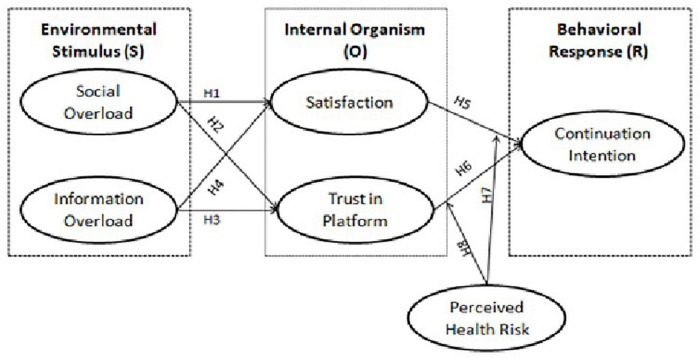
The theoretical framework of study.

User intentions have been extensively reviewed in the existing body of literature compared to tourism and related service areas (Dogru et al., 2020). The online platform continuation intention has been one of the widely studied areas in term of social media (Cao and Sun, 2018), but its implication in related areas and service platforms like tourism yet need further investigation by social scientists (Dogru et al., 2020). For instance, Jun’s (2020) study confirmed the role of perceived risk, brand credibility, and prior experience in user continuation intention. Wang et al. (2020) determined the role of user trust on continuation intention in light of social and technical perspectives of the Airbnb platform. The present study anticipates the negative association between information overload and social overload with continuation intention—similarly, the moderating role of perceived health risk considering the pandemic impact on tourism activities. In the end, this study combines the psychological, physical, and IT concepts. Figure 1, presents the theoretical framework.

### Stimuli-organism-response paradigm

We have adopted the SOR paradigm (Stimulus-Organism-Response) as an overreaching theoretical framework for this study. The literature confirms this framework’s predictive power in diverse management and marketing branches where the consumer is influenced by environmental stimuli (Hew et al., 2018; Nawaz et al., 2018; Chandra and Cassandra, 2019). SOR framework is incorporated from environmental psychology; the SOR framework considers the various environmental aspects that act as a stimulus (S) to influence the internal state of mind of respondents in terms of the organism (O) that eventually leads to the behavioral response (R). The framework explains how environmental stimuli in an outer environment of respondents fortify the inner state (Davis, 2001; Eroglu et al., 2001). Organism refers to inner feelings, beliefs, perceptions, and thinking (Bagozzi, 1986). Mehrabian and Russell (1974) describes it as the process of choice decision-making. The individual considers their ultimate choices and makes the final decision on these choices.

### Airbnb-based stimulus

Stimuli are the environmental determinants that an individual consumer encounters while experiencing different sets of products and services (Zhai et al., 2020). Airbnb-based stimuli considered for this study influence the internal psychological state and subsequent behavioral response. The Airbnb services provide an opportunity to interact, communicate, and co-create at a shared property (Hong and Lee, 2018; Tiamiyu et al., 2020; Jung et al., 2021). Recent literature supports the social and cognitive perspective of the online room booking service platforms (Ertz et al., 2018; Hong and Lee, 2018; Tiamiyu et al., 2020). The study of Sthapit et al. (2019) established the concept of information overload in the tourism segment, demonstrating how the excessive availability of information and choice influences the consumer satisfaction level and ultimately makes a negative contribution to continuity intentions.

The sharing platforms, such as Airbnb, provide additional opportunities to interact, develop, and maintain relationships in the real world, just like those in virtual networking sites (Yang and Lin, 2017). Such overload leads to multiple cognitive disorders like physical and social consequences, such as anxiety, lack of control, envy, depression, decision making, procrastination, and distrust (Lim and Yang, 2015; Andreassen et al., 2016; Meier et al., 2016; Alexander et al., 2018; Elhai et al., 2018). Social overload in virtual networks refers to the scenario in which platform users face extra demand for social support from other users in their social circle (Maier et al., 2015). Airbnb services help the visiting consumers to extend and maintain social circles, and this makes the Airbnb platform valuable for visitors to engage in social interaction (Lo, 2019). However, the Airbnb platform enhances the likelihood of social overload. For example, a consumer staying at an Airbnb host space tired of business meetings or tourist activities might receive too many social calls from other Airbnb guests in terms of emotional and material support, and this can develop a feeling of doing more for other guests causing social overload. Social overload is widely discussed in the existing body of literature, particularly in virtual networking sites (Nawaz et al., 2018; Lo, 2019). Some consumers might consider the Airbnb platform helpful in developing and maintaining a social circle, but continuous calls to maintain social interaction might decrease the value assessment of the Airbnb platform. So, the study proposes the following hypothesis:

H1: Social overload significantly contributes to consumer trust in the platform.

In the context of social overload, users are not tired of socialization, but they feel uneasy, overwhelmingly worried, and self-conscious about the social scene. This might lower user satisfaction with the service-providing platform. The existing body of literature provides evidence of virtual social overload consequences, such as emotional exhaustion (Maier et al., 2012). Emotional exhaustion also exists in physical and social interactions (McCarthy and Saegert, 1978). Emotional exhaustion represents the situation demanding continuous emotional engagement, leading to stress and lowering user satisfaction (Yang and Lin, 2017). Consumers opt for Airbnb host destinations for relaxation. When consumers receive continuous requests demanding social interaction and these requests are of high priority, demanding a prompt response, consumers stop relaxing and immediately respond to the social call. As the volume of such social requests is beyond the normality, a consumer might struggle to manage the situation, lowering user satisfaction with platform services. This situation represents the condition when social overload intervenes in emotional states. So, the study proposes the hypothesis as follows:

H2: Social overload makes a significant contribution to consumer service satisfaction.

Indeed, the excess information leads to multiple psychological consequences, such as the feeling of anxiety (Huang and Liaw, 2005), platform exhaustion (Luqman et al., 2017), technostress (Maier et al., 2015), and envy (Lim and Yang, 2015). Recent studies show that a huge amount of information influences the user’s trust in the platform (Mao et al., 2020). In the current context, the behavioral intention to use Airbnb is trust in a platform that measures the user continuation intention. Behavioral intention refers to user continuation intention (Wang et al., 2020). According to the TRA and trust theory, user behavioral intention is the primary factor in explaining user behavioral transactions (Mital et al., 2016; Paul et al., 2016). Consumers are more prompt to make business transactions with the platforms they trust (Mao et al., 2020). In the current context, information overload will negatively contribute to the user motivation (Zhang et al., 2020) to continue and might push them to look for alternative platforms. Thus, the study proposes the following hypothesis:

H3: Information overload significantly contributes to consumer trust in the platform.

The literature shows that excessive information processing may compromise user satisfaction with the product/service (Malhotra, 1982; Keller and Staelin, 1987). Moreover, excessive information makes the decision process more complicated as processing alternatives, removing some of them, and comparing alternatives will lead to cognitive strain (Sweller and Sweller, 2010). Thus, such a complex situation leads to cognitive overload and demands extra energy required to process the additional information, which eventually results in poor decisions and negative behavioral intention (Ryan et al., 2016). Users experiencing such informational overload are less satisfied with the service and their decisions (Tsiros and Mittal, 2000; Botti and Iyengar, 2004). This leads to the development of another hypothesis:

H4: Information overload makes a significant contribution to consumer service satisfaction.

### Organism and behavior response

The organism is the byproduct of stimuli, as they are influenced by environmental changes (Yangang, 2021). Prior literature on online room booking services confirms the critical role of service satisfaction (Chang C.-H. et al., 2014) and trust in the platform (Mao et al., 2020) during visitor stay. First, the level of service satisfaction results from service failure or success in meeting the user’s expectations (Liao et al., 2011). The second perspective is trust in the platform, defined as the level of behavioral dependency on a particular service (Wang et al., 2020). Consumers are more optimistic about repurchasing when they trust the platform (Mao et al., 2020). Higher satisfaction and higher trust in the platform enhance the consumer continuation intentions. Behavioral response is a blend of diverse factors (Ahe Ahn and Seo, 2018). The current study considers the consumer continuation intention in terms of service satisfaction and trust in the platform.

Consumer satisfaction is a blend of cognitive and emotional instigation or both (Oliver, 1997). In this study, we focus on cognitive processing, and in the context of satisfaction, it is considered a product of cognitive evaluation. The study by Mckinney et al. (2002) defines satisfaction as “an effective state representing an emotional response” while experiencing the service/product. Satisfaction enacts out of consumer post-consumption evaluation. Consumers have pre-purchase expectations and compare these expectations with post-consumption experiences (Oliver, 1980). This comparison leads to either positive disconfirmation or negative disconfirmation of consumer expectations. This study considers consumer satisfaction as an organism in response to stimuli, such as information and social overload. The literature has established that information and social overload adversely contribute to consumer’s internal mental state (Luqman et al., 2017). Considering these perspectives, this study proposes the following hypothesis:

H5: Service satisfaction makes a significant contribution to the user continuation intention.

Trust in the platform ensures consistent and competent behavior on account of both parties to ensure the consumer commitment to attaining the same benefits from such a relationship. In the present context, trust in the platform is influenced by the negative stimuli that might negatively contribute to the internal state of mind (trust in consumer platform: Airbnb). The prior research shows that in the SOR paradigm, the organism is the byproduct of the environmental stimuli (Chandra and Cassandra, 2019), and a similar relationship exists between the organism (trust in service platform) and response (continuation intention) (Hew et al., 2018). In the given scenario, the trust in the service platform negatively contributes to consumer continuation intention. Hence, the study proposes the following hypothesis:

H6: Trust in the service platform significantly contributes to consumer continuation intention.

### The moderating role of perceived health risk

In this era of a pandemic, the risk and tourism have become more closely interlinked, and in the case of sharing economy, the issue is more critical regarding the guest and host health. Jones et al. (2007) found that most tourists staying at shared residences are concerned about the health risk. Considering the complexity of tourism behavior, many question marks remain to be answered, including the extent to which perceived health risk remains an important indicator of tourism behavior (Jamrozy and Lawonk, 2017). Jonas et al. (2010) defined the health risk as a danger to tourist safety, security, and their host community. In this contest, COVID-19 is a risk to own health and security and also brings the host community at stack. A tourist already exposed to the cultural, psychological, physiological, emotional, and environmental challenges is burdened with health risks at an Airbnb host destination. Focusing on the pandemic situation, travel-related and health-related crises can be potentially answered through the qualitative study. So, the study proposes the hypothesis as follows:

H7: Perceived health risk significantly influences the relationship between consumer platform satisfaction and continuation intention.

H8: Perceived health risk significantly influences the relationship between consumer trust in the platform and continuation intention.

### The mediating role of the organism (service satisfaction and trust in the platform)

The SOR framework suggests an indirect effect of stimuli and behavioral response through organism theoretically. The existing body of literature provides ample justification for organisms mediating the relationship between stimuli and behavioral responses (Hew et al., 2018; Kim et al., 2020). In the present context, we consider that stimuli (information and social overload) negatively influence the internal state of the organism (service satisfaction and trust in the platform), ultimately leading to the adverse behavioral response (continuation intentions). This will be measured through the four mediation hypotheses.

H9a. Service satisfaction mediates the relationship between social overload and consumer continuation intention.

H9b. Service satisfaction mediates the relationship between information overload and consumer continuation intention.

H9c. Trust in the platform mediates the relationship between social overload and consumer continuation intention.

H9d. Trust in the platform mediates the relationship between information overload and consumer continuation intention.

## Methods

The purpose of this study is to investigate the role of the negative contribution of stimuli (S) to the internal organism (O) and the consequent behavior response (R).

### Questionnaire design

A structured questionnaire technique is adopted for data collection, and all the scale items are measured through the Likert 5-point scale [i.e., “strongly disagree (1) to strongly agree (5)”]. The measurement scales are adapted from existing literature that ensures the construct rationality. In particular, the five items for social overload are adapted from a previous study (Maier et al., 2015; Nawaz et al., 2018). The three items for information overload are adapted from Sthapit et al. (2019). The three items for satisfaction and four items of trust in the service platform are adapted from previous studies (Nawaz et al., 2018; Sthapit et al., 2019; Mao et al., 2020), respectively. The three items of continuation intentions are adapted from previous literature (Bhattacherjee, 2001; Maier et al., 2015). The three items for moderating variable perceived risk are adapted from Mao et al. (2020). The adapted 21 construct items and sources are given in the appendix. The adapted questionnaire is given in Table 1.

**TABLE 1:** Construct items and sources.

S. No.	Var.	Items	Statements	References
1	IO	IO1	There are too much information on Airbnb website that I am burdened in handling it.	Sthapit et al., 2019
		IO2	Because of too much information on Airbnb website, it is difficult to me to understand all of information.	
		IO3	I have no idea about where to find the information I needed on Airbnb website	
2	SO	SO1	I take too much care of my friends’ wellbeing on social networking sites.	Maier et al., 2015;
		SO2	I deal too much with my friends’ problems on social networking sites.	Nawaz et al., 2018
		SO3	My sense of being responsible for how much fun my friends have on social networking sites is too strong.	
		SO4	I am too often caring for my friends on social networking sites.	
		SO5	I pay too much attention to the posts of my friends on social networking sites.	
3	ST	ST1	I am satisfied with my Airbnb booking experience	Nawaz et al., 2018;
		ST2	Using Airbnb website is a pleasant experience	Sthapit et al., 2019
		ST3	Overall, I am satisfied with my Airbnb booking experience	
4	TinP	TinP1	As a platform, Airbnb cannot be trusted at all times	Pavlou and Gefen, 2004;
		TinP2	As a platform, Airbnb cannot be counted on to do what is right	Mao et al., 2020
		TinP3	As a platform, Airbnb has low integrity	
		TinP4	Airbnb is a incompetent platform	
5	PR	PR1	For me, using Airbnb when traveling involves considerable health risk	Pavlou and Gefen, 2004;
		PR2	For me, using Airbnb when traveling involves a high potential health risk for loss	Mao et al., 2020
		PR3	My decision to use Airbnb when traveling is health wise risky	
6	CI	SI1	I intend to switch to Airbnb accommodation service	Maier et al., 2015
		SI2	I will be looking to use Airbnb accommodation service for the future	
		SI3	I will stop using my current accommodation provider service	

IO, information overload; SO, social overload; SAT, satisfaction; TP, trust in platform; PR, perceived health risk; CI, continuance intention; Var, variable.

### Research development

The target population in this study comprises individuals having prior experience in ecotourism. To collect the data from the target population, the authors considered using the Google Form tool (online survey platform) for data collection. This method of data gathering is called the “purposive sampling method,” as this method allows the researcher to establish contact with the potential respondents and further request them to spread the questionnaire link to similar references (Malik et al., 2016; Dhir et al., 2018). The authors communicated to the respondents that their participation was voluntary and anonymized, assuring them their responses would be used only for academic purposes. The data were collected in September 2019 from Malaysian cities, namely, Kuala Lumpur, Penang, Putra Jaya, Johor Bahru, and Langkawi. The survey was limited to the Malaysian population with prior experience using Airbnb services. Table 2 provides the demographic details of the respondents. The authors consider Malaysia for this study for two reasons. First, Malaysia is a prime tourist spot in the far east, and second for being the highest tourist spending nation (Tourism Malaysia, 2021). Further, the country has more than 4 million foreign arrivals a year, earning 12 billion dollars from the tourism industry (Tourism Malaysia, 2021).

**TABLE 2:** Respondents profile.

Measures		Frequency	(%)
Gender	Male	153	50.5
	Female	150	49.5
Age	18–25	138	45.54
	26–33	62	20.46
	34–41	57	18.81
	42 and Above	46	15.18
Education	Secondary school	45	14.85
	Intermediate	55	18.15
	Graduation	180	59.41
	Post-graduation	23	07.59
Income	2000–3500	201	66.34
	3600–5000	55	18.15
	5100–Above	47	15.51

### Data analysis

The present study uses the partial least square (PLS) technique to investigate the hypothetical relationships through SmartPLS (Hair et al., 2016). PLS can provide a satisfactory result for both confirmatory and explorative studies, as it avoids the factor of determinacy. Moreover, PLS does not require theoretical support as considerable empirical support exists from well-established sources (Gefen et al., 2000). The alternative technique to conduct structural equation modeling (SEM) analysis is co-variance-based SEM (CB-SEM). The CB-SEM is good for theory testing, confirmation, and comparing alternative theories, and PLS-SEM is good for predicting internal relations and key constructs leading to relations (Hair et al., 2011). In short, the PLS-SEM technique was considered to study the relationship between stimuli (S) and organism (O), organism (O), and behavior response (R). Further, it evaluates the moderating role of perceived health risk and mediating role of organism (O) factors. To do so, the following analytical tools were considered:

a. SPSS 23 was implied to verify the quality of the data. The study results show the satisfactory data quality to proceed further with the PLS after assessing the missing data, outliers, and normality.
b. The study adapted SEM implied through the PLS (Hair et al., 2012). PLS is the second-generation technique used to test the measurement and structural model simultaneously along with the regression and confirmatory factor analysis (CFA).
c. SmartPLS 3.2.8 was implied for this study. The path analysis was conducted simultaneously with the moderating analysis, and mediation analysis was performed through the 80/20 rule.

## Results

### Profile of respondents

The survey is conducted with the help of Ph.D. candidates having prior experience. The target population consists of individuals having experience of online accommodation booking. At the successful completion of the questionnaire, respondents were offered a small gift. A total of 401 questionnaires were distributed; of them, 335 were collected and 32 were rejected later due to incomplete information. The remaining 303 questionnaires were used for final analysis. The demographic details are given in Table 1. The majority of respondents belong to the age group between 18 and 25 years, considering the fact that younger respondents are believed to be early adopters of technology, and contribute to 45.54% of the total respondents. Similarly, 20.46% fall in the age group of 26–33 years, 18.81% were in the age group ranging between 34 and 41 years, and 15.18 were above the age of 42 years. On the basis of education maximum, 59.41% of the respondents were university graduates, 18.15% had higher secondary certificate, and 14.85% were the secondary school graduates. Only 7.59% were post graduates. In terms of income, respondents were divided into three categories: 66.34% had a monthly income in the range of 2,000–3,500 RM, 18.15% had an income in the range of 3,600–5,000 RM, and 15.51% were found to have an income of 5,100 RM or above. The control variables, such as the gender, age, education, and income, were found to have non-significant impact.

### Results of measurement model

To measure construct reliability and validity, we conducted four sets of measurement procedures to determine internal consistency, convergent validity, and discriminant validity (Hair et al., 2011). We measure by using Cronbach’s alpha (α, which measures the internal consistency of items), factor loading (FL, which determines the load on structural members and connections), composite reliability (CR, which measures scale reliability), and average variance extracted (AVE, which measures variations in construct items). The confirmatory factor analysis (CFA) confirmed that all FL values were satisfactory and above the minimum value of 0.7. Similarly, the α-acceptable value was 0.7, which shows internal data consistency (Hair et al., 2012). The CR was above the threshold value of 0.7, and the AVE value was found to be above 0.5, which shows considerable validity as presented in Table 3. The convergent validity ensures that the constructs are internally different (Hair et al., 2011). Table 3 depicts that the square root values of AVE are above the correlation coefficients between variables, thus ensuring considerable discriminant validity.

**TABLE 3:** Construct validity.

S. No.	Constructs	Items	Loadings	α	CR	AVE	*R* ^2^	*Q* ^2^
1	Social overload (SO)	SO1	0.743	0.904	0.929	0.725		
		SO2	0.859					
		SO3	0.896					
		SO4	0.890					
		SO5	0.859					
2	Information overload (IO)	IO1	0.894	0.870	0.920	0.793		
		IO2	0.907					
		IO3	0.612					
3	Satisfaction (ST)	DS1	0.839	0.865	0.918	0.788	0.590	0.459
		DS2	0.911					
		DS3	0.911					
4	Trust-in-platform (TP)	TP1	0.793	0.869	0.911	0.719	0.647	0.454
		TP2	0.838					
		TP3	0.896					
		TP4	0.863					
5	Perceived risk (PR)	PR1	0.904	0.819	0.892	0.735		
		PR2	0.907					
		PR3	0.752					
6	Continuance intentions (SI)	CI1	0.914	0.851	0.910	0.771	0.660	0.481
		CI2	0.891					
		CI3	0.827					

Common method variance (CMV) is an important concern when data are gathered from a single source. To neutralize CMV concerns, this study performed multiple tests. First, Harman’s single-factor test is performed through exploratory factor analysis (EFA). All the items were distributed into 11 subgroups. The first factor explained 23.83% of the variance, which is within the limit of 40% (Podsakoff et al., 2011). Second, the 11-factor model is compared with the single-factor and two-factor models in SEM, wherein each factor contains three variables that represent the information that leads data to these constructs. The results of 11-factor model (*x*^2^ = 1,289.63, df = 781) are quite good compared to the single-factor model (*x*^2^ = 4,875.55, df = 291) and two-factor model (*x*^2^ = 8,547.63, df = 811) (Williams et al., 2010). In last, SEM-based marker variables have no theoretical link to any of the variables of this study (Siemsen et al., 2009). The outcome proved that the interaction between latent variables is not influenced by CMV. All these tests performed in this study proves that study is having no concern for CMV. Table 4 presents the discriminant validity.

**TABLE 4:** Measurement model and discriminant validity.

Variables	DS	IO	PR	SO	SI	TP
Satisfaction (ST)	**0.888**					
Information overload (IO)	0.712	**0.891**				
Perceived risk (PR)	0.554	0.468	**0.857**			
Social overload (SO)	0.667	0.620	0.461	**0.851**		
Continuance intention (SI)	0.703	0.654	0.523	0.586	**0.878**	
Trust in platform (TP)	0.762	0.778	0.486	0.642	0.732	**0.848**

SO, social overload; DS, satisfaction; IO, information overload; TP, trust in platform; PR, perceived risk; SI, continuance intentions.

Similarly, another way to ensure the non-existence of biasness in data is the HTMT criterion. The cut-off value of the HTMT is 0.90 (Henseler et al., 2014). Table 5 presents the output of HTMT for the current study, and it is quite clear that all the values are less than the cut-off value of 0.90, which ensures that the data are free from the issue of bias.

**TABLE 5:** Presenting the HTMT criterion.

Variables	DS	IO	PR	SO	SI	TP
Satisfaction (ST)	**0.898**					
Information overload (IO)	0.855	**0.891**				
Perceived risk (PR)	0.834	0.768	**0.857**			
Social overload (SO)	0.821	0.720	0.861	**0.851**		
Continuance intention (SI)	0.793	0.704	0.723	0.786	**0.878**	
Trust in platform (TP)	0.782	0.700	0.686	0.742	0.732	**0.848**

SO, social overload; DS, satisfaction; IO, information overload; TP, trust in platform; PR, perceived risk; SI, continuance intentions.

### Results of structural model

**FIGURE 2:**
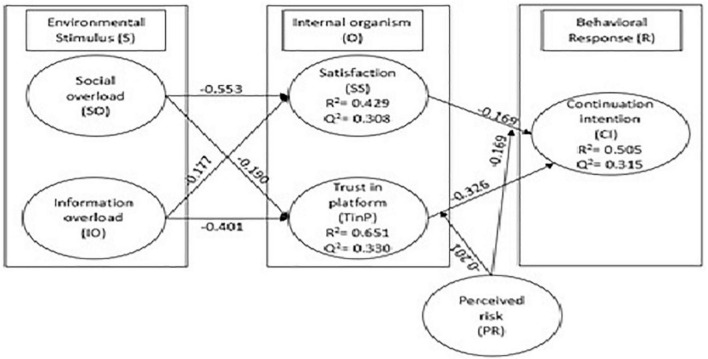
Presents the structural model of study.

To examine the testable statements, we evaluate path coefficients of the structural model. Figure 2 and Table 4 present the path coefficient values. Social overload negatively contributes to the service satisfaction, supporting H1 (β = −0.553, *p* < 0.001). Moreover, social overload also makes negative contribution to the trust in service platform, supporting H2 (β = −0.190, *p* < 0.001). Likewise, information overload makes negative contribution to service satisfaction and trust in platform, supporting H3 (β = −0.177, *p* < 0.001) and H4 (β = −0.401, *p* < 0.001). However, satisfaction also makes negative contribution to consumer continuation intentions, supporting H5 (β = −0.169, *p* < 0.001). Similarly, trust in service platform negatively contributes to consumer continuation intentions, supporting H6 (β = −0.326, *p* < 0.001).

In behavioral science studies, the standardized value of *R*^2^ of more than 0.2 is considered acceptable and standardized (Hair, 2016). As per the behavioral science standardization, the value of *R*^2^ more than 0.2 is recognized as satisfactory and acceptable. In the present study, the coefficient of determination value (*R*^2^) for satisfaction and consumer trust in the platform is 0.429 and 0.651, respectively. *R*^2^ for the continuation intentions of the consumer is 0.505, whereas the authors adopt the blindfolding procedure to examine the relevance of exogenous variables and the model performance, which is a simple reuse procedure (Chin, 1998; Mikalef et al., 2017). This procedure is a combination of function fit and cross-validation, and it further examines each construct’s predictive relevance by calculating changes in the criterion estimates (*Q*^2^) (Hair et al., 2012). A value of *Q*^2^ > 0 depicts the predictive relevance of the model. The results of the blindfolding procedure (*Q*^2^) followed in this study show that satisfaction (*Q*^2^ = 0.308) and consumer trust in the platform (*Q*^2^ = 0.330) and consumer continuation intentions (*Q*^2^ = 0.315) have satisfactory predictive relevance as they are observed above the cut of value.

### The moderation

The study evaluates the moderating relationship of perceived health risk between platform satisfaction, trust in the platform, and platform continuation intention. The study considers the negative moderating effect of perceived health risk between platform satisfaction, trust in the platform, and platform continuation intention. The study results show that perceived health risk negatively moderates the relationship between trust in the platform and continuation intention. So, hypothesis H8 is supported (H8: β = −0.201), whereas the insignificant relationship of perceived health risk is found between service satisfaction and continuation intention. Hence, hypothesis H7 is not supported (H7: β = −0.169). Table 6 presents hypothesis and moderation results.

**TABLE 6:** Hypotheses and moderation results.

Hyp.	Relationship	Std beta	Mean	*t*-value	*P*-value	Decision
H1	SO→ST	−0.177	0.365	4.847	0.000	Significant
H2	SO→TinP	−0.401	0.256	3.704	0.000	Significant
H3	IO→TinP	−0.190	0.620	9.744	0.000	Significant
H4	IO→ST	−0.553	0.489	6.440	0.000	Significant
H5	ST→SWI	−0.169	0.197	2.587	0.000	Significant
H6	TP→SWI	−0.326	0.383	4.890	0.000	Significant
**Moderation**						
H7	ST*PR*SI	−0.169	0.017	0.173	0.863	Insignificant
H8	TinP*PR*SI	−0.201	0.269	3.564	0.000	Significant

SO, social overload; ST, dissatisfaction; IO, information overload; TP, trust in platform; PR, perceived risk; CI, continuance intentions. * Shows the direction of relationship in moderation.

### The mediation

This study investigates the mediating role of the organism (user satisfaction with the service platform and trust in the platform) between the stimuli (information overload and social overload) and behavioral response. The study uses VAF (variance accounted for) to see the mediating influence of the organism between the stimuli and behavioral response. A VAF value less than 20% means that there is no mediation. A VAF value between 20 and 80% shows the existence of partial mediation. In the end, a VAF value above 90% depicts full mediation (Hair et al., 2014). Table 7 reveals that mediating hypotheses (H9a, H9b, H9c, and H9d) are partially mediating with an effect of 41.52, 30.23, 28.14, and 71.86, respectively, and all the VAF percentages are between 20 and 80%, confirming partial mediation.

**TABLE 7:** Variance accounted for (VAF) of the mediating variables (satisfaction and trust in platform).

Mediation								
**Hyp.**	**Relationship**	**Independent variable**	**Dependent variable**	**Mediating variable**	**Indirect effect**	**Total effect**	**VAF (%)**	**Decision**
H9a	SO→ST→SI	SO	SI	ST	0.071	0.171	41.52	Partial mediation
H9b	SO→Tin→PSI	SO	SI	TinP	0.101	0.334	30.23	Partial mediation
H9c	IO→ST→SI	IO	SI	ST	0.094	0.171	28.14	Partial mediation
H9d	IO→Tin→PSI	IO	SI	TinP	0.240	0.334	71.86	Partial mediation

SO, social overload; ST, satisfaction; IO, information overload; TP, trust in platform; PR, perceived risk; SI, continuance intentions.

## Discussion

This study evaluates the prominent role of stimuli (social overload and information overload), organisms (service satisfaction and trust in platform), and behavioral response (continuation intention) by integrating the mediating role of organisms between the stimuli and the response. Further, the study considers the moderating role of perceived health risk in terms of the shared economy platform to access the Airbnb user continuation intentions. Two types of environmental stimuli were measured in this study, that is, social and information overload. The results show that these two variables negatively influence the organism (service satisfaction and trust in the platform), which eventually influence the continuation intentions. The outcome validates the recent SOR-based studies, such as the study of Hew et al. (2018) found that user continuation intention in terms of mobile social tourism is influenced by the stimuli, such as perceived mobility, social presence, system, and service quality. Moreover, the study tests eight hypotheses, and the statistical outcome shows that most of the relationships are significant. The statistical results show that the users suffering social overload (at Airbnb shared accommodation) and information overload (at application) experience negative continuation intention and have a higher intention to leave the service platform permanently (Cao and Sun, 2018; Nawaz et al., 2018).

The social overload relationship between service satisfaction and trust in the platform is found to be inversely significant. The social overload makes a negative contribution to service satisfaction and trust in the platform. These findings are in line with the recent study of Cao and Sun (2018), which found the relationship between social overload contribution to a higher feeling of regret and SNS exhaustion. Further, the information overload also makes a negative contribution to service satisfaction and trust in the platform. Information overload influences the internal state of the user in multiple ways (Filieri et al., 2018; Sthapit et al., 2019). Similarly, this study found a negative relationship between information overload and service satisfaction, and trust in the platform. These findings of stimuli influencing the user’s internal state of mind are in line with SOR studies in tourism and related areas (Chandra and Cassandra, 2019; Kim et al., 2020; Zhai et al., 2020), whereas the organism makes a negative contribution to the user’s behavioral response (user continuation intention). This relationship is consistent with the recent finding of Zhang et al.’s (2020) study, which states that social fatigue leads to negative behavioral outcomes.

The negative relationship between the stimuli and organisms and later with the behavioral response shows that the prior studies have not considered the importance of service-related user internal state and have given more importance to the purely psychological and social constructs, such as regret, exhaustion, envy, crowding, and technostress (Cao and Sun, 2018; Nawaz et al., 2018). This study highlights the important role of service satisfaction and trust in the platform and the way these two can make a negative contribution to continuation intention while being influenced by the negative environmental factors.

**FIGURE 3:**
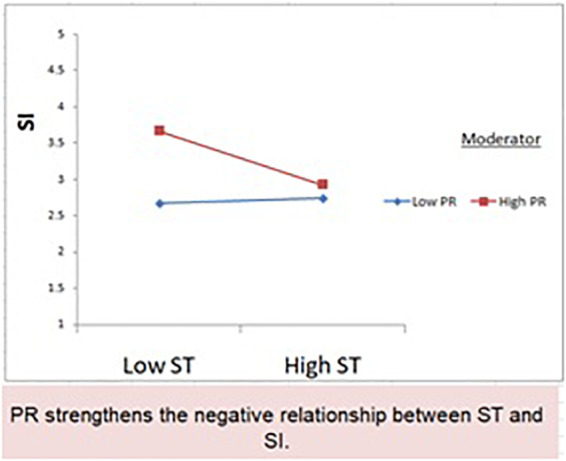
Presents the moderating relationship.

Further, the moderating role of perceived health risk between the organisms and the behavioral response is existing. Table 5 presents the moderation outcome. A significant relationship is found between the trust in the platform and the continuation intention. The perceived health risk strengthens the negative relationship as shown in Figure 3, whereas a non-significant relationship of perceived health risk was found between service satisfaction and continuation intention. The possible reason for this outcome can be the platform’s promise to provide swift and progressive health measures during the stay, but the situation on the property differs from the promised standardization. The user is more concerned with the actual measures taken at the property rather than the claims made at the online service platform. The literature provides support to the moderating claim of study, such as the study of Trivedi (2019) found inverse moderation of the perceived risk between the information quality, system quality, service quality, and customer experience in banking. Wang et al. (2019) found a positive relationship of perceived risk between the perceived value and willingness to use. Literature provides support for both positive and negative contributions of perceived risk.

Finally, service satisfaction and trust in the platform partially mediate the relationship between social overload, information overload, and the user continuation intention. The study results show that satisfaction has a direct and indirect relationship between social overload, information overload, and continuation intention. Similarly, the trust in the platform also, directly and indirectly, impacts the relationship between social overload, information overload, and user continuation intention. Both satisfaction and trust in the platform partially mediate the relationship between environmental stimuli and the behavioral response of the consumer. Further, the hypotheses are based on the SOR framework, that is, (O) mediates between the (S) and (R). This model has been prior involved with mediating studies and has given satisfying results. Chang (2017) used the framework to study the mediating role of perceived information utility and perceived social presence while studying the mediating role of media multi-tasking in impulse buying behavior. Moreover, Gil and Jacob (2018) adapted the model to study the mediating role of green satisfaction and green trust between the green purchase quality and green purchase intention. This study further enhances the boundaries of the SOR mediation literature from the tourism perspective.

## Conclusion

This study concludes the vital role of social overload and information overload in influencing the user’s internal state of mind consequently reducing continuation intention. The prior literature discussed the organisms in terms of social, psychological, and economic perspectives (Chandra and Cassandra, 2019; Kim et al., 2020), leaving a gap in terms of service-related perspectives. This study tries to fill this space. Furthermore, in this pandemic situation, the health-related perception of risk plays a vital role in opting for shared economic opportunities, such as Airbnb, where space would be physically shared among people. The moderating role of perceived health risk further enhances the study contribution. Before this, the literature focused on the general perception of perceived risk (Nawaz et al., 2018; Zhang et al., 2018). The study converts the focus to a more realistic perspective of perceived risk in terms of health.

### Implications

This section presents the study’s contribution to the existing body of literature and practical suggestions for managerial practice.

#### Theoretical implications

This study has some theoretical enlightenment. The statistical outcome of the research confirms the applicability of social overload, information overload, service satisfaction, trust in the platform, and the moderating role of perceived health risk in influencing the user continuation intention.

First, this study draws the researcher’s intention to the vital moderating role of perceived health risk in between user service satisfaction, trust in the platform, and user continuation intention. PLS-SEM results demonstrate that the perceived health risk moderates the relationship between service satisfaction and continuation intention. This existence of the inverse relationship shows the post-consumption concerns of the user and validates the gap between the platform claims and the actual service encounter experienced by the consumer.

Second, by integrating the SOR framework, the study extends the existing body of literature to the tourism industry. The framework stimuli (S) and behavioral response (R) are mediated by the user’s internal mental state known as an organism (O). SmartPLS results show a partial mediation of the organism. These results will open a new window for future research studies to conduct the mediating analysis in the context of tourism.

Third, the current study extends the concept of information overload and social overload to the tourist continuation intention of online platforms and their physical situation. This implies the role of overloads in the tourism industry and their ability to influence the service-related internal state of mind of the user.

In the end, the study highlights the vital post-consumption internal state of service satisfaction and user trust in the platform. These organisms play a prime role in user decision-making. The relationships discussed in this study provide many opportunities for business managers that will help them to revisit their existing set of strategies to retain and attract consumers.

#### Practical implications

The study has some practical implications for business managers and owners. First, the understanding of information technology factors that influence the user consumption process will help the local and multinational organizations (e.g., Airbnb) to restructure their strategies to enhance user satisfaction and trust to meet consumer needs and improve brand acceptability, such as putting some virtual checks on the host and web.

Second, the social overload at the host destination causes negative satisfaction with the service and service-providing platform. Recent studies show that the user is influenced by excessive socialization, and its related demand for impulsive response further adds social pressure (Nawaz et al., 2018; Lo, 2019). The business owners/managers can restrict the number of guests at the host destination through virtual checks of the number of bookings per day. In the Malaysian context, the number of hosts is growing at a quicker rate, and at the same time empty host destinations are increasing (Koenig, 2021). This gap provides a chance to overcome such experience and restrict per destination bookings.

Third, information overload is experienced in terms of the excessive information and number of choices offered that lead to a negative relationship with the service satisfaction and the trust in the platform. Recent information technology studies show that excessive information and choice lead to negative outcomes (Sthapit et al., 2019; Zhang et al., 2020). The business owners and managers can limit the information fallow on the virtual platform (application) and also the number of choices provided to a signal IP.

Furthermore, the business owners and the managers need to ensure that health-related hygienic measures are taken at the host destination. The literature provides ample evidence that consumer is concerned about health measures (Hassan and Soliman, 2021). The service providers must reduce the gap between the claims of the organization and the experience of the user. This will help to develop and maintain a longer service satisfaction and trust in the platform.

In short, social and information overload contributes negatively to consumer satisfaction and trust in the platform. This lead to reduced continuation motivation for the user, and the perceived health risk in an era of pandemic further reduces the user’s willingness to engage with the sharing economy platforms. Primarily, the business managers should focus on making the stay smooth and comfortable through the virtual restriction to control the number of people interacting at the host destination and at the same time controlling the excessive fallow of information and choices to the user.

### Study limitations and future direction

Just like other research studies, this study too has some limitations that may lead to a future opportunity for further research. First, the context of the study is limited to Malaysian consumers of Airbnb users only. Future studies can take a more aggressive approach and can perform multi-group analyses of multiple application users. Future studies can consider psychological and personality-related trades to fill the SOR framework to further enhance the existing body of literature in terms of the SOR framework and tourism industry. Moreover, this study adapted the measurement technique that collects the data at a single point in time. The futuristic studies can consider the longitudinal approach to validate the results. Furthermore, they can measure the moderating effect of philoxenia and fascination or some cultural constructs. The current study considers the PLS-SEM for data analysis, and further studies can use the fuzzy-set qualitative comparative analysis (fsQCA) for data analysis that might enhance the understanding of the concept.

## Data availability statement

The raw data supporting the conclusions of this article will be made available by the authors on request, without undue reservation.

## Ethics statement

Ethical review and approval was not required for the study on human participants in accordance with the local legislation and institutional requirements. The patients/participants provided their written informed consent to participate in this study.

## Author contributions

All authors listed have made a substantial, direct, and intellectual contribution to the work, and approved it for publication.
